# Parental Factors Associated With the Decision to Participate in a Neonatal Clinical Trial

**DOI:** 10.1001/jamanetworkopen.2020.32106

**Published:** 2021-01-12

**Authors:** Elliott Mark Weiss, Aleksandra E. Olszewski, Katherine F. Guttmann, Brooke E. Magnus, Sijia Li, Anita R. Shah, Sandra E. Juul, Yvonne W. Wu, Kaashif A. Ahmad, Ellen Bendel-Stenzel, Natalia A. Isaza, Andrea L. Lampland, Amit M. Mathur, Rakesh Rao, David Riley, David G. Russell, Zeynep N. I. Salih, Carrie B Torr, Joern-Hendrik Weitkamp, Uchenna E. Anani, Taeun Chang, Juanita Dudley, John Flibotte, Erin M. Havrilla, Charmaine M. Kathen, Alexandra C. O’Kane, Krystle Perez, Brenda J. Stanley, Benjamin S. Wilfond, Seema K. Shah

**Affiliations:** 1 Treuman Katz Center for Pediatric Bioethics, Seattle Children's Research Institute, Seattle, Washington; 2 Department of Pediatrics, University of Washington School of Medicine, Seattle; 3 Department of Pediatrics, Icahn School of Medicine at Mount Sinai, New York, New York; 4 Department of Psychology and Neuroscience, Boston College, Chestnut Hill, Massachusetts; 5 Department of Biostatistics, University of Washington School of Public Health, Seattle; 6 Division of Neonatology, Children’s Hospital of Orange County, Orange, California; 7 Departments of Neurology and Pediatrics, University of California San Francisco School of Medicine, San Francisco; 8 Department of Pediatrics, Baylor College of Medicine, San Antonio, Texas; 9 Department of Pediatrics, Mayo Clinic, Rochester, Minnesota; 10 Division of Neonatology, Department of Pediatrics, Children’s National Hospital, George Washington University School of Medicine and Health Sciences, Washington, DC; 11 Department of Neonatology, Children’s Minnesota Hospital, Minneapolis; 12 Department of Pediatrics, St Louis University School of Medicine, St Louis, Missouri; 13 Department of Pediatrics, Washington University School of Medicine in St Louis, St Louis, Missouri; 14 Department of Pediatrics, Cook Children’s Medical Center, Fort Worth, Texas; 15 Division of Neonatology, Cincinnati Children’s Hospital Medical Center, Cincinnati, Ohio; 16 Department of Pediatrics, Indiana University School of Medicine, Indianapolis; 17 Department of Pediatrics, University of Utah School of Medicine, Salt Lake City; 18 Department of Pediatrics, Vanderbilt University Medical Center, Nashville, Tennessee; 19 Department of Neurology, Children’s National Hospital, George Washington University School of Medicine and Health Sciences, Washington, DC; 20 Department of Pediatrics, Children’s Hospital of Philadelphia, Philadelphia, Pennsylvania; 21 Pediatrix Medical Group, San Antonio, Texas; 22 Department of Neurology, Children’s National Hospital, George Washington University School of Medicine and Health Sciences, Washington, DC; 23 Department of Pediatrics, Feinberg School of Medicine, Northwestern University, Chicago, Illinois

## Abstract

**Importance:**

It remains poorly understood how parents decide whether to enroll a child in a neonatal clinical trial. This is particularly true for parents from racial or ethnic minority populations. Understanding factors associated with enrollment decisions may improve recruitment processes for families, increase enrollment rates, and decrease disparities in research participation.

**Objective:**

To assess differences in parental factors between parents who enrolled their infant and those who declined enrollment for a neonatal randomized clinical trial.

**Design, Setting, and Participants:**

This survey study conducted from July 2017 to October 2019 in 12 US level 3 and 4 neonatal intensive care units included parents of infants who enrolled in the High-dose Erythropoietin for Asphyxia and Encephalopathy (HEAL) trial or who were eligible but declined enrollment. Data were analyzed October 2019 through July 2020.

**Exposure:**

Parental choice of enrollment in neonatal clinical trial.

**Main Outcomes and Measures:**

Percentages and odds ratios (ORs) of parent participation as categorized by demographic characteristics, self-assessment of child’s medical condition, study comprehension, and trust in medical researchers. Survey questions were based on the hypothesis that parents who enrolled their infant in HEAL differ from those who declined enrollment across 4 categories: (1) infant characteristics and parental demographic characteristics, (2) perception of infant’s illness, (3) study comprehension, and (4) trust in clinicians and researchers.

**Results:**

Of a total 387 eligible parents, 269 (69.5%) completed the survey and were included in analysis. This included 183 of 242 (75.6%) of HEAL-enrolled and 86 of 145 (59.3%) of HEAL-declined parents. Parents who enrolled their infant had lower rates of Medicaid participation (74 [41.1%] vs 47 [55.3%]; *P* = .04) and higher rates of annual income greater than $55 000 (94 [52.8%] vs 30 [37.5%]; *P* = .03) compared with those who declined. Black parents had lower enrollment rates compared with White parents (OR, 0.35; 95% CI, 0.17-0.73). Parents who reported their infant’s medical condition as more serious had higher enrollment rates (OR, 5.7; 95% CI, 2.0-16.3). Parents who enrolled their infant reported higher trust in medical researchers compared with parents who declined (mean [SD] difference, 5.3 [0.3-10.3]). There was no association between study comprehension and enrollment.

**Conclusions and Relevance:**

In this study, the following factors were associated with neonatal clinical trial enrollment: demographic characteristics (ie, race/ethnicity, Medicaid status, and reported income), perception of illness, and trust in medical researchers. Future work to confirm these findings and explore the reasons behind them may lead to strategies for better engaging underrepresented groups in neonatal clinical research to reduce enrollment disparities.

## Key Points

**Question:**

How do parents with infants enrolled in a neonatal clinical trial differ from those who decline participation?

**Findings:**

In this survey study including 269 parent responses, participants who enrolled their infant had different demographic characteristics (lower rate of Medicaid participation, higher income, less likely to identify as Black), reported their infant’s medical condition as more serious, and reported higher trust in medical researchers compared with those who declined to enroll. There was no association between study comprehension and enrollment.

**Meaning:**

Differences between parents who enrolled their infant and those who declined suggest potential targets for future research aiming to better engage underrepresented groups in neonatal clinical research.

## Teaser

This survey study compared parents who chose to participate in a neonatal clinical trial with those who declined, assessing differences in demographic characteristics, opinions on the severity of their child’s medical condition, and levels of trust in medical researchers.

## Introduction

Clinical trials are essential to advance neonatal care, but recruitment for them can be ethically complex. Because infants cannot provide consent for themselves,^1^ parents or guardians are asked for their permission.^2^ Recruitment can also be challenging because parents are under significant strain, and their vulnerable, sick infants often must be enrolled quickly if they are to participate.^3,4,5,6^

There are limited data to explain how parents decide whether to participate in neonatal clinical trials. Previous research has struggled to include the views of those who decline neonatal research^7,8^ with only a few exceptions.^9,10^ Low enrollment rates within neonatal clinical trials diminish the quality of data collected and limit the generalizability of results.^11^ Racial and ethnic disparities exist in neonatal research enrollment, but we do not fully understand which groups are overrepresented or underrepresented.^12,13^ Other parental factors that are potentially important in enrollment decisions include study comprehension^9,14^ and medical trust.^7,15,16^ Understanding these factors may enable researchers to improve recruitment processes, optimize enrollment rates, and decrease disparities in research participation.

The High-dose Erythropoietin for Asphyxia and Encephalopathy (HEAL) trial was a multisite randomized clinical trial (RCT) assessing erythropoietin as a neuroprotectant for infants with moderate to severe neonatal encephalopathy.^17^ Infants eligible for HEAL had an estimated gestational age greater than 36 weeks with moderate or severe neonatal encephalopathy undergoing therapeutic hypothermia. This population has a high risk of death in the neonatal period (approximately 28%) and of neurodevelopmental impairment among survivors (approximately 20%).^18^ We sought to identify differences between parents who enrolled and those who declined enrollment. We evaluated 4 categories: (1) infant characteristics and parental demographic characteristics, (2) parental perception of infant’s illness, (3) parental study comprehension, and (4) parental trust in clinicians and researchers.

## Methods

### Survey Development

We administered a survey to parents of infants approached for enrollment in the HEAL trial. The survey instrument was developed using: (1) validated question scales where available,^19,20^ (2) questions from previous surveys on parental participation in pediatric research,^9,16,21^ and (3) questions created after input from experts in survey design, medical decision-making, clinical trials, neonatology, and research ethics. We received feedback from the neonatal intensive care unit (NICU) parent advisory council at the University of Washington. This study followed American Association for Public Opinion Research (AAPOR) reporting guideline for best practices including survey design (ie, utilizing conceptual modeling, questionnaire pretesting), standardized administration, and clear reporting.^22^ Institutional review board (IRB) approval was received at each of the 12 participating sites and at subsites where applicable. Each IRB made the determination whether permission to participate was obtained by written informed consent, by oral assent, or if the survey was granted a waiver.

Infant characteristics included for analysis were birth weight, estimated gestational age at birth, whether inborn (recruited for HEAL at birth hospital) or outborn (transferred from another hospital), Medicaid status, severity of neonatal encephalopathy based on Sarnat exam prior to 6 hours of age,^23^ and day of life of survey. Parental demographic variables included gender, education, household income, prior experience with medical research, other children, race and ethnicity, and language spoken at home. Infant characteristics were obtained from the medical record; all other reported data were obtained via survey.

Parental perception of their infant’s condition was assessed using 7 novel questions. Responses were recorded on a 5-point Likert scale from 1 (“not at all”) to 5 (“very much”). Higher scores indicated increased concern of illness severity. Parental comprehension of the HEAL trial was assessed by 10 novel true-false questions. Questions addressed details of the HEAL trial, voluntariness of participation, and follow-up requirements. Trust was measured using 3 preexisting validated scales with minor adjustments in wording to fit the patient population: Hall’s 4-item trust in medical research scale^19^ and Dugan’s 5-item trust in the medical profession and 5-item trust in a physician scales.^20^

Surveys were refined using cognitive interviews with 10 parents of NICU patients by a single trained interviewer (E.M.W.) to assess comprehension and interpretation. The final survey was translated into Spanish by a certified medical translation service. Study data were collected and managed using REDCap electronic data capture tools (REDCap Consortium) hosted at the University of Washington.^24,25^ Both English and Spanish versions of the survey were inputted into the REDCap platform.

### Recruitment

Surveys were fielded at 12 of 17 HEAL sites from July 28, 2017, to October 30, 2019. All parents approached for HEAL trial enrollment were eligible. Parents of infants who were ineligible and those who were eligible but not enrolled for other reasons (eg, unavailability of study drug or research coordinator) were excluded. Given the acute stressors of neonatal encephalopathy diagnosis and deciding on enrollment in a clinical trial, eligibility did not begin until the infant’s fifth day of life. Each family could choose 1 parent to participate until their infant’s hospital discharge or death.

Interviewers read questions aloud to parents and entered responses directly into REDCap to minimize transcription errors. Interviewers were excluded from surveying families they had approached for HEAL RCT participation. Preference was given for in-person administration, but the survey was administered over phone when necessary. After survey completion, parents were offered a gift card or small toy based on local IRB preference. A Spanish language version of the survey was offered to families using a Spanish language speaking interviewer or via interpreter, if necessary.

### Variables

We combined self-reported race and ethnicity into 4 mutually exclusive categories: Hispanic, non-Hispanic Black (hereafter “Black”), non-Hispanic White (hereafter “White”), and non-Hispanic other (ie, Asian, American Indian or Alaska Native, Native Hawaiian or other Pacific Islander), as has been done previously and is consistent with the US Census Bureau approach.^26^

To simplify analysis, we dichotomized the 5-point responses for each question set (eg, for parental perception of infant’s illness, 1-3 was categorized as “less concerned” and 4-5 as “concerned”).

### Statistical Analysis

Standard descriptive statistics were applied to our factors of interest. We fit several multivariate logistic regression models to investigate the associations of parental factors (ie, demographic characteristics, perception of infant’s illness, study comprehension, and trust scales) with enrollment decision. We examined the association between each parental factor and enrollment decision by comparing odds ratios (ORs) between groups. In each model, we adjusted for race, Medicaid status, and household income to eliminate potential confounding. We report estimates of ORs along with 95% confidence intervals constructed using robust standard errors.

Data were analyzed October 2019 through July 2020. All *P* values are 2-sided and not adjusted for multiple comparisons; an association was considered significant if *P* < .05. We performed a planned secondary analysis to evaluate the interaction between race/ethnicity and each of the parental factors separately to assess whether each factor was differentially associated with enrollment by race. All analyses were conducted using the R statistical package version 3.6.1 (R Project for Statistical Computing).

## Results

### Infant Characteristics and Parental Demographics

Analysis included 269 of 387 (69.5%) eligible parents across 12 US sites who completed the survey. Of these, 177 (67.0%) participants were mothers, 121 (45.7%) were receiving Medicaid, and 124 (48.1%) reported income $55 000 or greater. Self-reported race/ethnicity of participants was 148 (55.0%) White, 61 (22.9%) Hispanic, and 39 (14.5%) Black (Table 1). Surveys captured 183 of 242 (75.6%) HEAL-enrolled and 86 of 145 (59.3%) HEAL-declined parents. The 86 parents who declined HEAL and participated in our survey included 1 who did not respond after approach by study staff, 16 who did not wish to talk to study staff, and 69 who declined trial participation after discussion with study staff. There were no significant differences in infant characteristics between the 2 groups (Table 1). Parents of infants who were not receiving Medicaid or who reported higher income were significantly more likely to enroll their children in the study (eg, Medicaid recipients vs annual income ≥$55 000 enrollment: 74 (41.1%) participants vs 94 (52.8%) participants; Table 1). Enrollment status was significantly different between race/ethnicity groups, with lowest relative enrollment among Black parents compared with White parents (OR, 0.35; 95% CI, 0.17-0.73). In a planned secondary analysis, none of the interactions between race/ethnicity and other parental factors were significant.

**Table 1.** Infant Characteristics and Parental Demographic Characteristics

Characteristics	Participants, No. (%)		
	All	Enrolled	Declined
Total^a^	269	183 (68.0)	86 (32.0)
Birth weight, mean (SD), g	3409 (631)	3434 (602)	3356 (688)
Estimated gestational age, mean (SD), wk	39.1 (1.4)	39.1 (1.4)	39.0 (1.5)
Inborn	46 (17.1)	32 (17.5)	14 (16.3)
Severe encephalopathy	46 (17.1)	30 (16.4)	16 (18.6)
Infant day of life on survey, mean (SD)	9.5 (7.8)	9.0 (7.2)	10.4 (8.8)
Age of respondent, mean (SD), y	29.6 (6.4)	29.9 (6.4)	29.0 (6.3)
Mother respondent	177(67.0)	120 (66.3)	57 (68.7)
Medicaid recipient^b^	121(45.7)	74 (41.1)	47 (55.3)
Education			
Some high school or high school graduate	82 (30.8)	52 (28.6)	30 (35.7)
Some college	59 (22.2)	39 (21.4)	20 (23.8)
≥College graduate	125 (47.0)	91 (50.0)	34 40.5)
Income ≥$55 000/y^b^	124(48.1)	94 (52.8)	30 (37.5)
Past research experience	42 (15.7)	28 (15.3)	14 (16.7)
Other children	106 (39.7)	71 (38.8)	35 (41.7)
With chronic health problems	8 (7.5)	8 (11.3)	0
Who had been in NICU	14 (13.2)	10 (14.1)	4 (11.4)
English spoken at home	240 (90.2)	164 (90.1)	76 (90.5)
Survey administered in English^c^	257 (96.6)	174 (96.1)	83 (97.6)
Race/ethnicity^b^			
Hispanic	61 (22.9)	43 (23.6)	18 (21.4)
Non-Hispanic			
Black	39 (14.5)	19 (10.4)	20 (23.3)
White	148 (55.0)	108 (59.0)	40 (46.5)
Asian	10 (3.7)	7 (3.8)	3 (3.5)
American Indian or Alaska Native	6 (2.2)	4 (2.2)	2 (2.4)
Native Hawaiian or Other Pacific Islander	2 (0.7)	1 (0.5)	1 (1.2)
Other	2 (0.7)	1 (0.5)	1 (1.2)

Abbreviation: NICU, neonatal intensive care unit.

^a^ Percentages are calculated that exclude missing data in the denominator.

^b^ *P* value for Medicaid status = .04; for reported income, *P* = .03; and for race, *P* = .04; all other *P* values not significant.

^c^ Surveys could be administered in English only at 1 site and in English or Spanish at 11 sites.

### Perception of Infant’s Illness

We observed significant differences in parental perception of their infant’s condition. Parents who enrolled their infant in HEAL reported their infant’s medical condition to be more serious compared with parents who declined for 4 of 7 questions (eg, enrollment and agreement with the statement, “I very much or moderately thought my infant’s illness was a serious medical condition,” ≥4 on a 5-point scale: OR, 5.7; 95% CI, 2.0-16.3) (Table 2). After adjusting for race, Medicaid status, and household income, the association between parental perception of illness and enrollment remained significant. The association between parental perception of their infant’s illness and study enrollment did not differ by race, reported income, or Medicaid status.

**Table 2.** Parental Perception of Infant’s Condition

I very much or moderately…^a^	Participants, No. (%)			Odds ratio (95% CI)^b^
	All	Enrolled	Declined	
… thought my infant’s illness would affect his or her life	187 (69.8)	145 (79.2)	42 (49.4)	3.6 (2.0-6.5)
… thought my infant was sick	207 (77.5)	148 (81.3)	59 (69.4)	1.8 (0.9-3.4)
… was concerned about my infant’s illness	258 (96.3)	181 (98.9)	77 (90.6)	8.4 (1.6-43.2)
… understood my infant’s illness	127 (47.4)	85 (46.4)	42 (49.4)	0.9 (0.5-1.5)
… thought my infant’s illness was a serious condition	245 (92.8)	175 (96.7)	70 (84.3)	5.7 (2.0-16.3)
… thought my infant’s illness would have long term effects	186 (69.7)	141 (77.0)	45 (53.6)	2.7 (1.5-5.0)
I thought my infant was likely to die at birth^c^	133 (50.8)	95 (53.7)	38 (44.7)	1.3 (0.7-2.3)

^a^ Number (%) of participants responding 4 or 5 on 5-point scale from 1 (not at all) to 5 (very much).

^b^ Odds ratio is that of enrollment in HEAL comparing a participant who selected 4 or 5 with those who selected 1 to 3 on the first 6 questions, and yes to no for last question. Ratios are adjusted for race, Medicaid status, and household income.

^c^ No. (%) of participants answering “yes.”

Because we found this association between parental perception of illness and enrollment, we looked for an association between parental perception of illness and Sarnat classification. The percentage of parents who responded that they “very much” or “moderately” thought their infant was sick was higher for parents of infants with severe Sarnat stage (41 of 46 [89%]) compared with parents of infants with moderate Sarnat stage (166 of 221 [75%]) (OR, 3.2; 95% CI, 1.2-11.0) (eTable in the Supplement). For each of the other 6 questions related to parental perception of their infant’s illness, the association with Sarnat classification was not significant.

We asked a single question on parental self-assessment of understanding of their infant’s illness: “How much did you understand your infant’s illness?” Self-assessment of understanding of their infant’s illness did not differ between parents who enrolled in HEAL and those who declined. Roughly half of respondents self-reported a low level of understanding of their infant’s condition.

### Comprehension of HEAL Study

Parental comprehension was high, with a mean (SD) of 8.9 (1.2) out of 10 questions answered correctly; the median (interquartile range) number of correct answers was 9.0 (8.0-10.0). Total correct answers did not differ significantly between those who did and did not enroll in HEAL (Table 3).

**Table 3.** Study Comprehension

Characteristics	Correct responses, No. (%)			*P* value
	All (n = 269)	Enrolled (n = 183)	Declined (n = 86)	
All infants who joined HEAL would receive Epo	229 (87.4)	164 (90.1)	65 (81.2)	.07
Epo has never been used in infants before	223 (86.1)	160 (87.9)	63 (81.8)	.27
Infants who joined HEAL would have a 50:50 chance of receiving placebo or Epo	248 (94.7)	176 (96.7)	72 (90.0)	.05
Infants with brain injury at birth are at high risk of long-term neurologic problems	242 (92.0)	169 (92.3)	73 (91.2)	.96
The purpose of the HEAL study is to find out if Epo can protect infants with brain injury at birth from developing neurologic problems	255 (97.0)	176 (96.7)	79 (97.5)	>.99
Infants in the HEAL study may have more blood drawn for analysis	220 (84.6)	157 (85.8)	63 (81.8)	.53
Infants in the HEAL study will get more blood transfusions	227 (88.3)	165 (90.7)	62 (82.7)	.11
Doctors know that Epo can protect infants with brain injury at birth from having neurologic problems	166 (63.8)	108 (60.0)	58 (72.5)	.07
Participation in the HEAL study was optional	263 (99.6)	182 (99.5)	81 (100)	1.0
Infants in the HEAL study are followed through age 2 y	244 (93.8)	176 (96.2)	68 (88.3)	.03
Total score correct answers, mean (SD)	8.9 (1.2)	9.0 (1.3)	8.7 (1.2)	.05

Abbreviation: Epo, erythropoietin; HEAL, High-dose Erythropoietin for Asphyxia and Encephalopathy.

### Trust of Clinicians and Researchers

Enrolled parents reported significantly higher levels of trust in medical researchers compared with parents who declined (mean [SD] score, 71.7% [17.2%] vs 64.8% [20.8%]) (Table 4). There was no difference in reported trust in the medical profession (61.7% [13.7%] vs 60.1% [13.0%]) or trust in the infant’s neonatologist between groups (70.3% [11.6%] vs 70.1% [11.1%]). After adjusting for race, Medicaid status and household income, the association of trust in medical researchers with enrollment decision remained significant (mean difference, 5.3%; 95% CI, 0.3%-10.3%). The association between trust and study enrollment did not differ by race, reported income, or Medicaid status.

**Table 4.** Parental Trust

Survey prompt	Score, mean (SD)			Mean difference (95% CI)^d^
	All	Enrolled	Declined	
Trust in medical researchers^a^	69.5 (18.7)	71.7 (17.2)	64.8 (20.8)	5.3 (0.3 to 10.3)
Trust in medical profession^b^	61.2 (13.5)	61.7 (13.7)	60.1 (13.0)	1.5 (−2.1 to 5.1)
Trust in your infant’s neonatologist^c^	70.3 (11.4)	70.3 (11.6)	70.1 (11.1)	−0.3 (−3.5 to 2.9)

^a^ Using Hall’s 4-item trust in medical research scale^19^ with mean (SD) score indexed to a 100-point scale.

^b^ Using Dugan’s 5-item patient trust in the medical profession scale^20^ with mean (SD) score indexed to a 100-point scale.

^c^ Using Dugan’s 5-item trust in a physician scale^20^ with mean (SD) score indexed to a 100-point scale.

^d^ Adjusted for race, Medicaid status, and household income.

## Discussion

This study lends insight into factors associated with parental decision-making that can inform future strategies to improve enrollment for neonatal RCTs. First, rates of participation differed by race and income. Second, although Sarnat classification of objective illness severity did not differ between groups, parents who declined HEAL enrollment felt their infant was less ill than those who enrolled. Third, there was no association between study comprehension and enrollment. Fourth, a specific decreased trust in medical researchers may be driving lower enrollment rates, rather than general medical distrust.

### Infant Characteristics and Parental Demographic Characteristics

We found lower enrollment among parents on Medicaid and with lower incomes. These findings are consistent with previously observed recruitment disparities in adults from underserved populations.^27^ In pediatrics, the relationship between socioeconomic status (SES) and research enrollment is poorly described, and with conflicting results.^10,16,28^ We also found that Black parents enrolled their infants in HEAL less frequently than White parents. The association between the parental factors we studied (perception of their infant’s illness, medical trust, and study comprehension) and HEAL enrollment did not differ by race, Medicaid status, or reported income.

Black participants are the most underrepresented within US research in adults.^29,30^ While some have emphasized historical abuse and persistent distrust in medical research,^31^ others have emphasized limited access to clinical trials.^32^ Other factors that may contribute to disparities in research participation include implicit and explicit biases of research team members and financial or transportation barriers.^33,34,35^ Establishing community partnerships and building relationships with underrepresented populations are also important.^27^ Enrollment of racial and ethnic minorities in pediatric clinical research, though less well-studied, is lower than for White children including within critical care,^28,36^ oncology,^37^ and neonatology.^11,12^ Conversely, assessments of published articles in 3 general pediatric journals found overrepresentation of Black participants and underrepresentation of Hispanic participants when comparing with US Census Bureau data.^13,38^ The lack of clarity highlights the need for more work in this area.

Contributors to disparities in clinical trial participation are complex and will require multilevel solutions.^39^ Racism and poverty are both social determinants of health that profoundly impact pediatric health outcomes.^40,41^ Neonatal outcomes in the US are worse for Black infants^42,43,44,45,46,47^ and for infants from families with low SES.^48^ Populations that are systematically underrepresented in neonatal research fail to benefit from research, which could exacerbate existing disparities in neonatal clinical outcomes.^45^ Future work must study these disparities and other potential drivers of differences in enrollment to improve representation within neonatal research and address persistent injustices.

### Parental Perception of Infant’s Condition

We found that parents who enrolled their infants in HEAL reported perceiving their infant’s medical condition to be more serious compared with those who declined HEAL enrollment. Although we do not have additional data describing illness severity, the Sarnat scores^23^ in the 2 groups were equivalent. This suggests that the groups were comprised of infants with similar illness severity at time of HEAL enrollment. Thus, the most likely reason for this finding is that parents were more likely to decline HEAL because of an incorrect assessment that their child was less sick than they were. Believing an infant to be gravely ill might make a parent more willing to enroll in research.^49,50^ Importantly, all infants eligible for HEAL were at high risk for neurodevelopmental impairment.

Future work should evaluate how illness perception may influence enrollment decisions. If parental perception of illness severity mediates the decision to enroll, then an intervention that improves parental understanding of their infant’s medical condition may both improve the quality of clinical decision-making and increase enrollment for neonatal clinical trials.

Future work should evaluate the contributors to parental perception of their infant’s illness. Such work must gather more granular clinical data to better understand how parents develop their perception of their infant’s illness. Considering the HEAL eligible population, for example, it would be useful to know short-term (eg, intubation or pressor requirement), medium-term (eg, MRI findings, neurological exam, and feeding support requirements at discharge), and long-term (eg, 2-year neurodevelopmental assessment) outcomes, in addition to how perception of illness may change over time.

### Study Comprehension

Although study comprehension is an enduring challenge for clinical trial enrollment^51^ and has been posited as a reason for lower enrollment of racial and ethnic minorities and individuals of lower SES,^9,14^ we did not find differences in study comprehension between parents who enrolled and those who did not enroll in neonatal research. Researchers were able to educate parents to achieve high levels of comprehension about the HEAL study. This suggests that study comprehension was not a major problem among parents approached for the HEAL trial. Future work must assess the role of study comprehension for enrollment decisions in other neonatal clinical trials.

### Trust

We found higher levels of trust in medical researchers among enrolled parents compared with parents who declined, but no difference in trust in clinicians on 2 trust scales. The higher trust in medical researchers among enrolled parents is consistent with reflections of experienced NICU clinical researchers^15^ as well as interview^7^ and survey^16^ studies of parents considering research participation.

Our findings are novel because they add nuance to the existing literature on the relationship between trust and parental decision to enroll in pediatric research.^7,16^ Our findings suggest it is not simply global trust in medicine, but a particular kind of trust—that of medical researchers—which may mediate enrollment decisions. There has been growing attention to race/ethnicity concordance between participants and research staff as part of research engagement with underrepresented populations.^52^ The relationship between concordance, trust in medical researchers, and research participation remains poorly described. For example, a 2020 study^53^ found greater clinical trial attrition among race/ethnicity concordant dyads compared with discordant dyads. Such findings, paired with the pragmatic challenges of implementation,^54^ highlight the complexity of these issues. Future work should assess whether interventions to build trust in researchers and other study personnel, whether at the community or individual level, can increase enrollment for neonatal clinical trials.

### Limitations

This study had several limitations. Although we succeeded in including a large number who declined participation in HEAL, families who declined survey participation might differ from those who participated. Characteristics of the HEAL eligible population and of the HEAL study may have influenced the enrollment decision in unknown ways that may impact generalizability. We did not assess recruitment practices (eg, tracking of potential participants, research team composition, method of contact with families), which varied across sites and may influence enrollment.^55^ Our eligibility window (infant’s fifth day of life through hospital discharge) may have affected recall.^56^ Although we asked parents to answer questions thinking back to the time of enrollment, responses may have been influenced by subsequent events. We did not assess trust in research institutions, which may be important and different than trust in medical researchers.^57^ We did not evaluate race/ethnicity concordance between parents and research staff. Our questions about illness perception, based on previous work suggesting parental perception of illness severity and urgency influences clinical decision-making in the NICU,^58,59^ have not been validated. Our study comprehension questions may not have been at the right level of difficulty to capture the full range of parental understanding.

## Conclusions

This study identified the following factors associated with whether a parent enrolled their child in a neonatal clinical trial: demographic characteristics (ie, reported parental race/ethnicity, infant’s Medicaid status, and reported parental income), perception of infant’s illness severity, and trust in medical researchers. We found lower rates of enrollment among Black parents compared with White parents, parents with lower income compared with those with higher income, parents who perceived their infant as less severely ill, and parents with lower trust in medical researchers. Future work should seek to confirm our findings in other neonatal clinical trials and explore the reasons behind them. In particular, greater understanding of how parents evaluate their infants’ clinical condition and how this influences clinical trial enrollment is critically important. Future work should also assess strategies for better engaging underrepresented groups in neonatal clinical research and reducing enrollment disparities. Understanding the differences between parents who enroll and those who do not enroll their infant in neonatal clinical trials will help identify interventions to improve enrollment and ensure that the results and benefits of research are more accurate and equitable.

## Notes

Supplement. **eTable.** Perception of Severity of Childs’ Illness and Sarnat Classification
