# Supplementary material for: Parental Factors Associated With the Decision to Participate in a Neonatal Clinical Trial
Source: JAMA Netw Open. 2021 Jan 12;4(1):e2032106. doi: 10.1001/jamanetworkopen.2020.32106 (PMC7804922; doi:10.1001/jamanetworkopen.2020.32106)
Supplement: Supplement. — eTable. Perception of Severity of Childs’ Illness and Sarnat Classification [file jamanetwopen-e2032106-s001.pdf]

Supplemental Online Content

Weiss EM, Olszewski AE, Guttmann KF, et al. Parental factors associated with the decision to participate in a neonatal clinical trial. *JAMA Netw Open*. 2021;4(1):e2032106. doi:10.1001/jamanetworkopen.2020.32106

eTable. Perception of Severity of Childs' Illness and Sarnat Classification

This supplemental material has been provided by the authors to give readers additional information about their work.

© 2021 Weiss EM et al. *JAMA Network Open.*

eTable. Perception of Severity of Childs' Illness and Sarnat Classification

I very much or moderately …	Moderate[a]	Severe	Odds Ratio (95% CI)[b]
… thought my infant's illness would affect his or her life[b]	154 (69%)	33 (72%)	1.5 (0.7, 3.4)
… thought my infant was sick	166 (75%)	41 (89%)	3.2 (1.2, 11)
… was concerned about my infant's illness	213 (96%)	45 (98%)	--[c]
… understood my infant's illness	108 (49%)	19 (41%)	0.8 (0.4, 1.6)
… thought my infant's illness was a serious condition	201 (92%)	44 (96%)	4.7 (0.9, 87)
… thought my infant's illness would have long term effects	149 (67%)	37 (80%)	2.4 (1.0, 6.0)
I thought my infant was likely to die at birth[c]	105 (49%)	28 (61%)	1.6 (0.8, 3.4)

[a]The columns are Sarnat severity staging of participant's infant.
[b]The odds ratio has adjusted for Medicaid status, household income, and race.
[C]Not calculated because only a single participant with an infant in the severe category did not choose "very much" or "moderately."

© 2021 Weiss EM et al. *JAMA Network Open.*
